# Efficacy and Safety of Pyronaridine–Artesunate for the Treatment of Uncomplicated *Plasmodium falciparum* and *Plasmodium vivax* Malaria in Myanmar

**DOI:** 10.4269/ajtmh.20-0185

**Published:** 2020-06-08

**Authors:** Kay Thwe Han, Khin Lin, Zay Yar Han, Moe Kyaw Myint, Kyin Hla Aye, Aung Thi, Badri Thapa, Maria Dorina Bustos, Isabelle Borghini-Fuhrer, Pascal Ringwald, Stephan Duparc

**Affiliations:** 1 Department of Medical Research, Ministry of Health and Sports, Yangon, Myanmar;; 2 Department of Medical Research (Pyin Oo Lwin Branch), Ministry of Health and Sports, Pyin Oo Lwin Township, Myanmar;; 3 National Malaria Control Programme, Department of Public Health, Ministry of Health and Sports, Nay Pyi Taw, Myanmar;; 4 World Health Organization, Yangon, Myanmar;; 5 World Health Organization, Bangkok, Thailand;; 6 Medicines for Malaria Venture, Geneva, Switzerland;; 7 World Health Organization, Geneva, Switzerland

## Abstract

Four single-arm, prospective, clinical studies of pyronaridine–artesunate efficacy in uncomplicated *Plasmodium falciparum* or *Plasmodium vivax* malaria were conducted in Myanmar between 2017 and 2019. Eligible subjects were aged at least 6 years, with microscopically confirmed *P. falciparum* (*n* = 196) or *P. vivax* mono-infection (*n* = 206). Patients received pyronaridine–artesunate once daily for 3 days with follow-up until day 42 for *P. falciparum* or day 28 for *P. vivax*. For the primary efficacy analysis, adequate clinical and parasitological response (ACPR) in the per-protocol population at day 42 for *P. falciparum* malaria was 100% (88/88; 95% CI: 95.9, 100) in northern Myanmar (Kachin State and northern Shan State), and 100% (101/101; 95% CI: 96.4, 100) in southern Myanmar (Tanintharyi Region and Kayin State). *Plasmodium falciparum* day-3 parasite clearance was observed for 96.9% (190/196) of patients. Mutations in the *P. falciparum* Kelch propeller domain (*K13*) were detected in 39.0% (69/177) of isolates: F446I (14.7% [26/177]), R561H (13.0% [23/177]), C580Y (10.2% [18/177]), and P574L (1.1% [2/177]). For *P. vivax*, the day-28 ACPR was 100% (104/104; 95% CI: 96.5, 100) in northern Myanmar and 100% (97/97; 95% CI: 96.3, 100) in southern Myanmar. Across both *P. vivax* studies, 100% (206/206) of patients had day-3 parasite clearance. There were no adverse events. Pyronaridine–artesunate had excellent efficacy in Myanmar against *P. falciparum* and *P. vivax* and was well tolerated. This study supports the inclusion of pyronaridine–artesunate in national malaria treatment guidelines for Myanmar.

## INTRODUCTION

The effective treatment of malaria is a priority health target in Myanmar, and the country has committed to eliminating falciparum malaria by 2025 and all kinds of malaria by 2030. Case management is used in areas targeted for elimination, and rapid diagnostic testing and treatment with artemisinin-based combination therapy (ACT) are provided free of charge by public and private clinics. The current first-line agent for *Plasmodium falciparum* malaria is artemether–lumefantrine, followed by single-dose primaquine (0.75 mg/kg). Dihydroartemisinin–piperaquine or artesunate–mefloquine are available as alternative ACT regimens. *Plasmodium vivax* malaria is treated with chloroquine complemented by radical curative treatment with primaquine (0.25 mg/kg/day for 14 days).

Myanmar is one of the six countries with known artemisinin-resistant *P. falciparum*.^1^ The artemisinin resistance phenotype is defined as an increase in day-3 parasite positivity following ACT treatment, that is, a delay in parasite clearance.^1,2^ Clinical efficacy is not affected unless resistance also develops to the partner drug, but this is more likely to emerge and to spread once the antimalarial activity of the artemisinin component is compromised. Unfortunately, multidrug-resistant *P. falciparum* is a serious problem across the Greater Mekong Subregion (GMS).^3,4^ Thus, potential alternative therapeutic options for Myanmar require evaluation in case current first-line treatments for falciparum malaria become ineffective, as observed in Cambodia.^3,4^

The ACT pyronaridine–artesunate has shown good efficacy for uncomplicated falciparum and vivax malaria in large-scale clinical trials conducted in Asia and Africa.^5–12^ Within the GMS, pyronaridine–artesunate PCR-corrected day-42 adequate clinical and parasitological response (ACPR) rates against falciparum malaria in western Cambodia were 87.9% (95% CI: 80.6, 93.2) in 2014–2015,^13^ but more recently > 98% efficacy was noted for this region (2018)^14^ and efficacy was > 96% in eastern Cambodia (1997),^15^ and > 96% in Vietnam (2017–2018).^16^ Thus, pyronaridine–artesunate can remain a valuable treatment option for uncomplicated falciparum malaria in regions where the utility of other antimalarial drugs has been compromised by drug resistance.

Intensive malaria control and treatment efforts in Myanmar have reduced the prevalence of *P. falciparum*.^17^ However, *P. vivax* remains refractory to current interventions and has become the dominant parasite in some areas.^18,19^ Pyronaridine–artesunate is the first ACT specifically registered for *P. vivax* malaria, with demonstrated high clinical efficacy across Southeast Asia.^12,14^ Chloroquine resistance has been reported in Myanmar,^20–23^ and to plan effective *P. vivax* elimination programs, the efficacy of alternative therapies requires confirmation.

This study assessed the efficacy of pyronaridine–artesunate for the treatment of uncomplicated *P. falciparum* and *P*. *vivax* malaria in southern and northern Myanmar to support a review of the national malaria treatment policy and to inform the design of malaria elimination programs in the context of artemisinin resistance.

## MATERIALS AND METHODS

### Study design and ethics.

**Figure 1.**
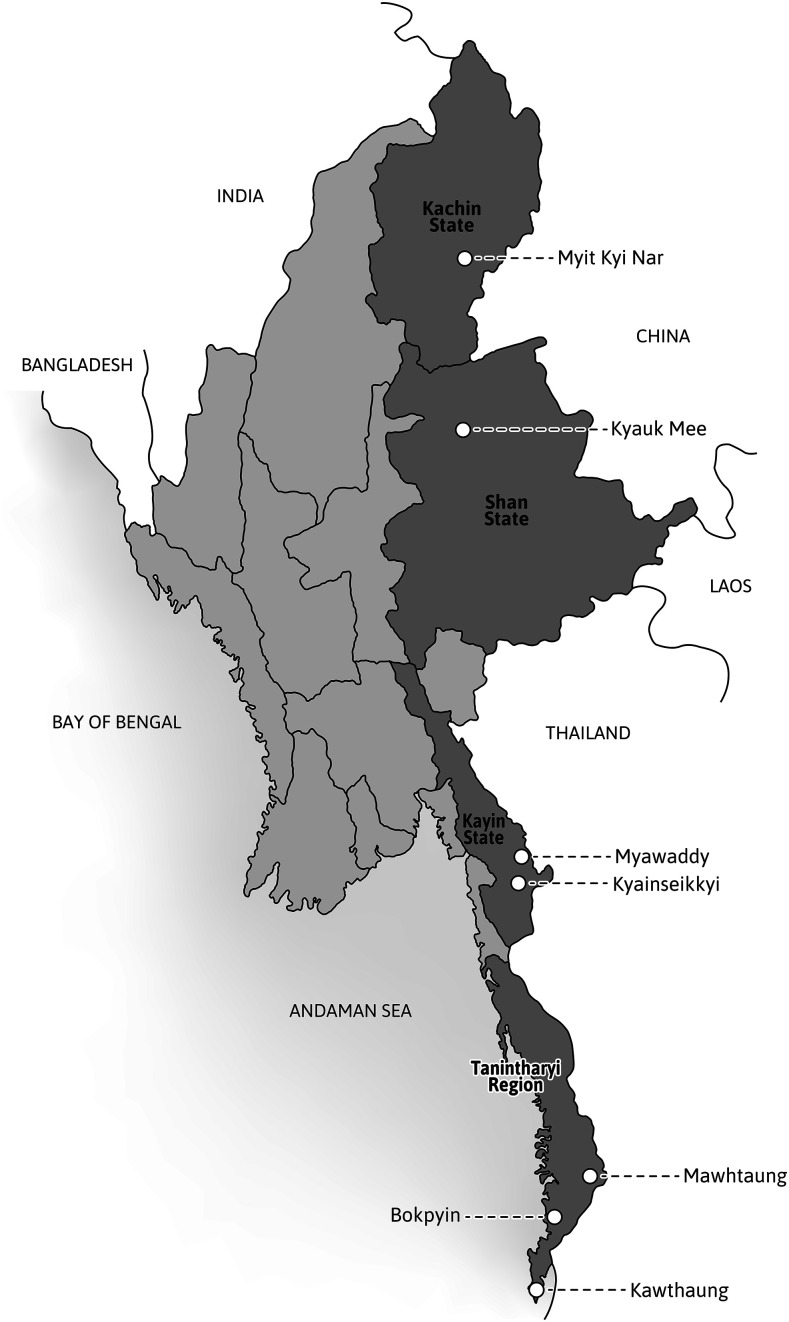
Study sites.

Four single-arm, prospective, antimalarial treatment efficacy studies were conducted between July and December 2017 in two townships in northern Myanmar (Kachin State and northern Shan State) and between August 2017 and November 2019 in five townships in southern Myanmar (Tanintharyi Region and Kayin State) (Table 1, Figure 1).

**Table 1:** Overview of four surveillance studies evaluating pyronaridine–artesunate therapeutic efficacy conducted in Myanmar during 2017–2019 and patient baseline characteristics

Description/characteristic	*P. falciparum*		*P. vivax*	
	Northern Myanmar	Southern Myanmar	Northern Myanmar	Southern Myanmar
Study registration*	ACTRN12618001952235	ACTRN12618001620213	ACTRN12618001952235	ACTRN12618001621202
Study sites	Kachin State	Tanintharyi Region	Kachin State	Tanintharyi Region
	(Myit Kyi Nar)	(Kawthaung and Mawhtaung)	(Myit Kyi Nar)	(Kawthaung and Bokpyin)
	Northern Shan State	Kayin State	Northern Shan State	Kayin State
	(Kyauk Mee)	(Myawaddy and Kyainseikkyi)	(Kyauk Mee)	(Myawaddy and Kyainseikkyi)
Dates conducted	July 1, 2017 to December 25, 2017	August 9, 2017 to November 3, 2018	July 1, 2017 to September 30, 2017	May 2, 2018 to November 2, 2019
Treatment	Pyronaridine–artesunate once daily for 3 days plus single-dose primaquine (0.75 mg/kg)	Pyronaridine–artesunate once daily for 3 days plus single-dose primaquine (0.75 mg/kg)	Pyronaridine–artesunate once daily for 3 days plus primaquine (0.25 mg/kg/day for 14 days)	Pyronaridine–artesunate once daily for 3 days plus primaquine (0.25 mg/kg/day for 14 days)
Number of patients	90	106	104	102
Male:female	58:32	70:36	69:35	65:37
Mean age (years) (SD) (range)	30.1 (12.4) (9–61)	30.3 (14.5) (6–59)	33.2 (11.2) (10–60)	21.0 (13.9) (6–58)
Age ≥ 16 years, *n* (%)	78 (86.7)	88 (83.0)	92 (88.5)	55 (53.9)
Age 5–15 years, *n* (%)	12 (13.3)	18 (17.0)	12 (11.5)	47 (46.1)
Mean weight (kg) (SD) (range)	50.2 (10.7) (22–64)	49.1 (10.5) (20–65)	47.4 (7.4) (27–60)	61.8 (9.9) (44–76)
Mean geometric parasitemia (range)	8,014 (510–37,200)	8,051 (989–92,472)	2,481 (750–9,854)	1868 (266–18,466)

*P. falciparum* = *Plasmodium falciparum*; *P. vivax* = *Plasmodium vivax*.

* Registered on the Australian New Zealand Clinical Trials Registry; owing to an administrative error, ACTRN12618001952235 and ACTRN12618001620213 were registered retrospectively. Note that two of the studies were registered under a joint protocol (ACTRN12618001952235).

The studies complied with the Declaration of Helsinki, Good Clinical Practice guidelines, and all relevant local and international laws. The protocol was reviewed and approved by the Ethics Review Committee, Department of Medical Research, Ministry of Health & Sport, Myanmar, and the WHO Research Ethics Review Committee. All adult participants and parents or guardians of participants aged < 18 years provided written informed consent; in addition, assent was required from children aged ≥ 12 years. The trials were registered on the Australian New Zealand Clinical Trials Registry (Table 1). The standard WHO therapeutic efficacy test protocol was used for all four studies, and all were reported using the same methods and analysis.^24^

### Participants.

Across all four studies, eligible patients had to be at least 6 years of age, have microscopically confirmed uncomplicated malaria with fever or history of fever, be able to swallow oral medication, and be willing to comply with the study protocol. Patients had to have microscopically confirmed mono-infection with *P. falciparum* (500–100,000/µL asexual parasites) or microscopically confirmed *P. vivax* mono-infection (parasitemia of 250–100,000/µL asexual parasites) (Table 1).

General exclusion criteria were signs and symptoms of severe falciparum malaria,^25^ body weight < 20 kg, mixed *Plasmodium* infection, severe malnutrition, the presence of febrile conditions other than malaria or other underlying chronic or severe diseases, regular medication which might interfere with antimalarial pharmacokinetics, a history of hypersensitivity reaction, or contraindications to the study medicine. All female participants aged 12–17 years were excluded because pregnancy testing before enrollment was not feasible owing to cultural conditions. Otherwise, women of child-bearing age were excluded if they had a positive pregnancy test, were breastfeeding, or were unwilling to use contraception. In addition, if patients had *P. vivax* infection, they were excluded if their hemoglobin was < 8 g/dL (using HemoCue^®^, Ängelholm, Sweden).

### Treatment.

Pyronaridine–artesunate tablets (180:60 mg pyronaridine–artesunate; Pyramax^®^, Shin Poong Pharmaceutical Co., Ansan, Republic of Korea) were given orally once daily for 3 days (days 0, 1, and 2). Dosing was according to the body weight; 20 to < 24 kg, one tablet per day, 24 to < 45 kg, two tablets per day, 45 to < 65 kg, three tablets per day, and ≥ 65 kg, four tablets per day. Patients were treated as outpatients, but all doses were supervised and patients were observed for 30 minutes after dosing. In case of vomiting, patients were re-dosed and if the patient vomited again, they were withdrawn and administered rescue medication. Patients with *P. falciparum* malaria also received single-dose primaquine (0.75 mg/kg). In the case of *P. vivax* malaria, after testing for glucose-6-phosphate dehydrogenase (G6PD) deficiency using Carestart™ rapid diagnostic test (AccessBio, Somerset, NJ), primaquine was administered according to national treatment guidelines, that is, 0.25 mg/kg/day for 14 days if the patient was G6PD normal or 0.75 mg/kg/day once weekly for 8 weeks if they were G6PD deficient.

### Procedures.

Patients were treated for 3 days (days 0, 1, and 2), with follow-up on days 3, 7, 14, 21, 28, 35, and 42 for patients with *P. falciparum* and up to day 28 for those with *P. vivax*. The medical history, including prior and concomitant medication, and demographic information were collected at baseline. Physical examination and clinical assessment were conducted on day 0 before dosing, on days 1, 2, and 3, and at all follow-up visits. Giemsa-stained thick and thin blood films for parasite identification and counts were examined on day 0 and thick blood films were examined on days 1, 2, 3, and at all follow-up visits or any day the patient returned. Asexual parasites were enumerated using standard methods.^26^ The slides were read by two qualified microscopists and parasite densities calculated as the average of the two counts. Discordant results (differences of > 50%) were examined by a third independent microscopist and parasite density calculated as the average of the two closest counts. In the case of *P. falciparum* recurrence, polymerase chain reaction genotyping (PCR) was used to distinguish between recrudescence and reinfection by comparing *P. falciparum* genes *msp1*, *msp2*, and *glurp* in baseline samples versus those obtained at failure, using published methods.^27^ Throughout the follow-up, patients were routinely asked about old symptoms and new symptoms emerging since the previous visit. The nature and incidence of adverse events and serious adverse events were recorded throughout the study.

### Molecular surveillance methods.

Blood drops were collected on filter paper for assessment of molecular markers of artemisinin resistance in the Kelch propeller domain (*K13*). Studies were conducted at the laboratory of the Department of Medical Research, Yangon, using published methods.^28^ Briefly, nested PCR was used to amplify *P. falciparum K13* genes (codon 432–703) from extracted DNA samples, and purified PCR products were sequenced using a 3500× L genetic analyzer (ABI Applied Biosystems, Foster City, CA). Sequence analysis of the *K13* gene was performed using Sequencher^®^ software (version 5.4, Gene Codes Corporation, Ann Arbor, MI) and BioEdit sequence alignment editor software (version 7.2.5, Informer Technologies, Inc., Roseau Valley, Dominica). The *K13* propeller gene sequence for *P. falciparum* 3D7 was retrieved from GenBank (ID; AL844509.2) and used as a reference strain. Fifteen percent of *K13* mutant samples and 5% of wild-type samples were randomly selected and sent to the Pasteur Institute for external quality control; the results were found to be 100% concordant.

### Outcomes.

Efficacy outcomes were based on an assessment of the parasitological and clinical outcome of antimalarial treatment according the WHO guidelines.^26^ Patients were classified as having early treatment failure, late clinical failure, late parasitological failure, or ACPR, defined as absence of parasites without previous treatment failure. Adequate clinical and parasitological response was determined at day 42 for *P. falciparum* and day 28 for *P. vivax*. Safety evaluations were the incidence of any adverse event occurring during the study period.

### Statistical methods.

Efficacy was evaluated using two methods, by calculating the proportion of patients with ACPR in a per-protocol analysis and by Kaplan–Meier estimation of the cumulative incidence of ACPR over the study period. Patients lost to follow-up or withdrawn from the study were excluded from the per-protocol analysis or censored in the Kaplan–Meier analysis at the last available visit. In the PCR-adjusted analysis, patients were excluded or censored if the PCR results were unclassifiable or if the results of PCR indicated that the failure was due to reinfection.

For each study, the estimated treatment failure rate for pyronaridine–artesunate was < 5%. For a confidence level of 95% and a precision around the estimate of 5%, a minimum of 73 patients was required. Allowing for 20% withdrawals, patients lost to follow-up, and reinfection, the recruitment target was 88 patients per study.

## RESULTS

### Patients.

In northern Myanmar, 90 patients were treated for *P. falciparum* malaria, with two lost to follow-up and 104 for *P. vivax* with none lost to follow-up. In southern Myanmar, 106 patients were treated for *P. falciparum* malaria, five of which were lost to follow-up, and 102 patients were treated for *P. vivax* malaria, five of which were lost to follow-up. Patient baseline characteristics are shown in Table 1. The majority of patients with *P. falciparum* were male (65.3% [128/196]) and older than 16 years (84.7% [166/196]). For *P. vivax*, most patients were male (65.0% [134/206]) and 71.4% (147/206) were older than 16 years.

### Efficacy.

For patients with *P. falciparum* in northern Myanmar, day-42 ACPR with pyronaridine–artesunate was 100% (88/88; 95% CI: 95.9, 100) in the per-protocol analysis and 100% in the Kaplan–Meier analysis (Table 2). In southern Myanmar, *P. falciparum* day-42 ACPR was 100% (101/101; 95% CI: 96.4, 100) in the per-protocol analysis and 100% using Kaplan–Meier analysis (Table 2). As there were no cases of recurrence, PCR adjustment was unnecessary and PCR-adjusted ACPR was the same (100% in both regions). Across all enrolled patients from northern Myanmar (*n* = 90), the day-3 parasite positivity rate was 6.7% (6/90; 95% CI: 2.5, 13.9). However, all of the patients with parasites at day 3 were from Kachin State, giving a day-3 parasite positivity rate of 13.3% (6/45; 95% CI: 5.1, 26.8) for this region. All of the 106 enrolled patients from southern Myanmar had parasite clearance at day 3.

**Table 2:** Pyronaridine–artesunate efficacy against *P. falciparum* and *P. vivax* in Myanmar, per-protocol analysis

Population and outcome	*P. falciparum*		*P. vivax*	
	Northern Myanmar	Southern Myanmar	Northern Myanmar	Southern Myanmar
Patients recruited (*n*)	90	106	104	102
Lost to follow-up (*n*)	2	5	0	5
Per-protocol population (*n*)	88	101	104	97
Adequate clinical and parasitological response (%) (*n*/*N*) (95% CI)	100 (88/88) (95.9, 100)	100% (101/101) (96.4, 100)	100 (104/104) (96.5, 100)	100 (97/97) (96.3, 100)

*P. falciparum* = *Plasmodium falciparum*; *P. vivax* = *Plasmodium vivax*. Adequate clinical and parasitological response was evaluated at day 42 for *P. falciparum* and day 28 for *P. vivax*.

For *P. vivax*, pyronaridine–artesunate day-28 ACPR in the per-protocol analysis was 100% (104/104; 95% CI: 96.5, 100) in northern Myanmar and 100% (97/97; 95% CI: 96.3, 100) in southern Myanmar. In both regions, ACPR was 100% when estimated using Kaplan–Meier analysis. All 206 enrolled patients across both studies had parasite clearance by day 3.

### Artemisinin resistance molecular surveillance.

**Figure 2.**
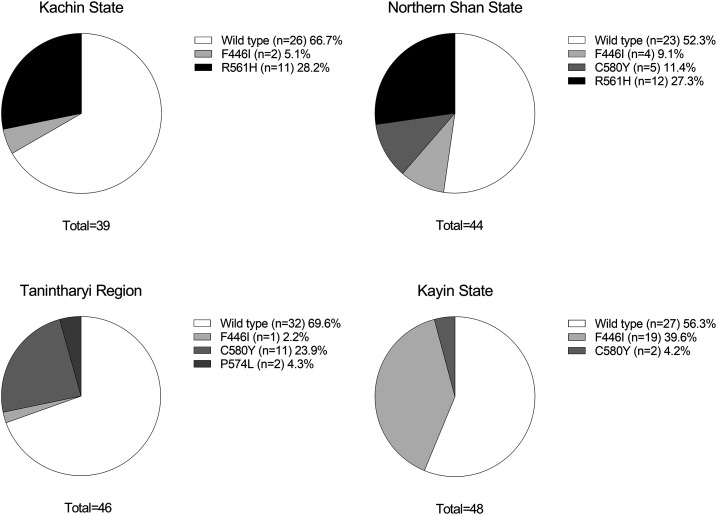
Prevalence of *Plasmodium falciparum K13* mutations in isolates collected in 2017 from northern Myanmar (Kachin State and northern Shan State) and in 2017/2018 from southern Myanmar (Tanintharyi Region and Kayin State).

*Plasmodium falciparum K13* sequences were obtained from 92.2% (83/90) of isolates collected in 2017 in northern Myanmar (Figure 2). The proportion of isolates with *K13* mutations was 41.0% (34/83); 33.3% (13/39) in Kachin State and 47.7% (21/44) in northern Shan State. In both states, the most common mutant was *K13*(R561H), with an overall prevalence of 27.7% (23/83). The C580Y mutant was only detected in northern Shan State (11.4% [5/44]).

For 2017/2018 data from southern Myanmar, *K13* sequences were available for 88.7% (94/106) of *P. falciparum* isolates (Figure 2). Overall, the *K13* mutation rate was 37.2% (35/94); 30.4% (14/46) in Tanintharyi Region and 43.8% (21/48) in Kayin State. *K13*(C580Y) was the most common mutant in Tanintharyi Region, whereas F446I was the dominant mutant in Kayin State (Figure 2).

As pyronaridine–artesunate clinical efficacy was 100%, no correlation could be made between *K13* mutations and efficacy. Parasitemia at day 3 was only detected in six patients from Kachin State; isolates from two patients had the *K13*(R561H) mutation, but the other four isolates were *K13* wild type. Thus, there was no relationship between *K13* mutations and day-3 parasite positivity rate.

### Safety.

There were no adverse events of any cause recorded following treatment with pyronaridine–artesunate in either *P. falciparum*- or *P. vivax*-infected patients.

## DISCUSSION

Malaria elimination in Myanmar is critically dependent on the continued efficacy of ACTs against *P. falciparum*. However, artemisinin-resistant *P. falciparum* is present in the region, and there is the potential for the emergence of partner drug resistance.^29,30^ Thus, the situation is fragile, and the impressive progress that Myanmar has made in reducing malaria prevalence and mortality over the last decade could be easily reversed should multidrug-resistant *P. falciparum* become established. Once a parasite emerges with a clear survival advantage under intense selective pressure from a narrow range of drug therapies, a selective sweep can cause a rapid loss of efficacy. This was seen for *P. falciparum* in Cambodia with a dominant artemisinin/piperaquine multidrug-resistant haplotype emerging within months of dihydroartemisinin–piperaquine being introduced as first-line therapy.^3^ The most recent data from the GMS indicate that pyronaridine–artesunate retains good efficacy in Cambodia and Vietnam against multidrug-resistant *P. falciparum*,^14–16^ and the current study confirms excellent efficacy in Myanmar. These findings are reassuring, potentially allowing the diversification of antimalarial therapy, and suggesting an alternative option should resistance undermine the efficacy of currently recommended antimalarial drugs.

Molecular surveillance of *P. falciparum K13* mutations suggests a genetically independent artemisinin-resistant parasite population in Thailand–Myanmar–China region versus Cambodia–Laos–Vietnam.^1,2,31^ An extensive geospatial mapping study reported the proportion of *K13* mutants as 47.6% for Kayin State, 37.1% for Kachin State, and 66.7% for Shan State.^32^ This is broadly consistent with our findings (43.8%, 33.3%, and 47.7%, respectively). In the current study, four different *K13* mutants were found: F446I, C580Y, and P574L in southern Myanmar and F446I, C580Y, and R561H in northern Myanmar. All of these are validated artemisinin resistance markers. However, in the current study, the majority of patients were aparasitemic by day 3, and delayed parasite clearance was associated with R561H in two cases and wild-type *K13* in four cases. These findings are consistent with 2014–2015 data from Rakhine, Shan, and Kachin states, where artemether–lumefantrine and dihydroartemisinin–piperaquine retained high efficacy (> 96% and 100%, respectively), despite the presence of artemisinin-resistant parasites in Shan and Kachin states.^33^ As seen in the current study, the day-3 parasite positivity rate was low following artemether–lumefantrine (0–3.6%), and all parasites were cleared with dihydroartemisinin–piperaquine.^33^

These studies indicate that in Myanmar, the circulating *K13* mutations have limited impact on parasite clearance at present. This is in contrast to Cambodia–Laos–Vietnam, where a multidrug-resistant haplotype conferring high-level resistance to artemisinin and piperaquine has reached near fixation in some regions.^1,3,14–16^ Correspondingly, day-3 parasite positivity rates are relatively high, for example, 35.6–46.7% in eastern regions of Cambodia and 28.6–41.7% in the west.^14,15^ Moreover, high clinical failure rates are observed for dihydroartemisinin–piperaquine.^4^ A limitation of this study was that molecular markers of *P. falciparum* resistance to piperaquine and mefloquine were not investigated. However, there is currently no clinical evidence of drug failure for ACT regimens containing these antimalarial drugs in Myanmar.^34^

The antimalarial resistance situation in Myanmar requires continued close monitoring. However, maintaining effective surveillance in a low-transmission setting is challenging. Finding eligible patients with *P. falciparum* malaria in southern Myanmar was difficult, requiring two transmission seasons to achieve target recruitment. Falciparum malaria prevalence was low in the study areas, with only 1.6% (119/7,457) of patients screened having microscopically confirmed falciparum malaria. Social factors also hampered recruitment, for example, migrant workers in the Myanmar–Thai border area were fearful and reluctant to engage, and a clinic set up by a nongovernmental organization also treated malaria patients in the region.

As the prevalence of *P. falciparum* has declined, *P. vivax* has emerged as a significant barrier to malaria elimination in Myanmar.^19,35^ There is also evidence of *P. vivax* chloroquine resistance in Myanmar,^20–23^ including high-level resistant strains circulating in northeast Myanmar.^22^ Although the prevalence of these strains is thought to be low, the success of elimination efforts based on chloroquine/primaquine could be jeopardized and intensification of *P. vivax* antimalarial treatment may further select and propagate resistant strains. Pyronaridine–artesunate had 100% efficacy in the current study against *P. vivax*. This is consistent with data from Cambodia, Thailand, India, and Indonesia showing that pyronaridine–artesunate was non-inferior to chloroquine, but with more rapid parasite and fever clearance.^12^ Thus, pyronaridine–artesunate presents a clinically validated alternative to chloroquine for the treatment of *P. vivax* malaria and is the only ACT to receive approval for this indication by a stringent regulatory authority (European Medicines Evaluation Agency).^12^

Pyronaridine–artesunate is known to cause asymptomatic mild-to-moderate transient increases in liver transaminases in some malaria patients.^5,8^ A limitation of the current study was that hepatic enzyme levels were not monitored. The standard protocol for therapeutic efficacy studies does not require laboratory monitoring, although there is provision for specific safety measures to be included if required. However, a recent extensive investigation showed no increase in the risk of hepatic transaminase elevations on repeated pyronaridine–artesunate dosing, and no clinical manifestations of hepatic injury associated with the biochemical observations.^6,7^ There were also no clinical indications of any hepatic adverse effect of pyronaridine–artesunate in the current study.

In conclusion, this study evaluated pyronaridine–artesunate efficacy against *P. falciparum* and *P. vivax* malaria in Myanmar. There were no cases of treatment failure for either parasite, and the therapy was well tolerated. Pyronaridine–artesunate could be added to the national malaria treatment guidelines in Myanmar and can be considered as an additional tool for programmatic decisions aimed at achieving malaria elimination for both *P. falciparum* and *P. vivax*.
